# Treg: tolerance *vs* immunity

**DOI:** 10.18632/oncotarget.4648

**Published:** 2015-06-25

**Authors:** Moanaro Biswas, Cox Terhorst, Roland W. Herzog

**Affiliations:** Department of Pediatrics, University of Florida, Gainesville, FL, USA

**Keywords:** Immunology Section, T cells, dendritic cells, regulatory T cells

**Figure 1:**
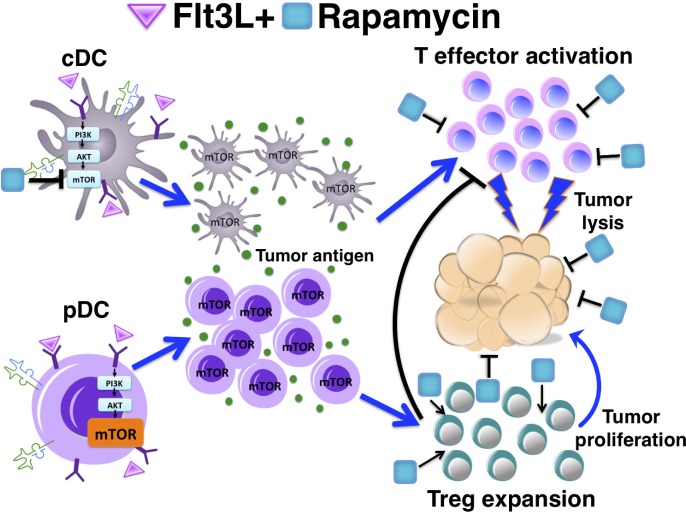
Effect of drug combination and dosing on tolerance induction and proposed implications for cancer immunotherapy Rapamycin inhibits tumor cells, while Flt3L expands DC (which may improve T effector cell activation but also increase Treg). However, intermediate doses of rapamycin induce Treg; and combination with Flt3L preferentially expands pDC, thereby further increasing Treg. Differences in mTOR signaling among T cell and DC subsets are in part responsible for these differential effects.

Regulatory T cells (Treg) are requisite for the prevention of autoimmunity, transplant rejection, and tolerance to therapeutic protein delivery. Dendritic cells (DCs) are crucial for FoxP3^+^ Treg homeostasis, and their expansion can increase Treg numbers. By contrast, Treg are a hindrance to anti-cancer therapy as they suppress immune responses to tumors. Interestingly, some of the same drugs and molecular targets that are being explored for cancer immunotherapy also promote induction and expansion of FoxP3^+^ Treg. For example, DCs are critical for the activation of effector T cells, and expansion of DCs may therefore activate and/or expand T cells with specificity to tumor antigens. We recently combined a cytokine-mediated expansion of DC with inhibition of mTOR, the mammalian target of the immunosuppressant rapamycin, to promote immune tolerance, which was accompanied by an increase of Treg cells [1]. Interestingly, the drugs used in this regimen (rapamycin and FMS-like tyrosine kinase 3, Flt3L/CD135) are also used in cancer immunotherapy. As the concentrations of rapamycin that inhibit mTOR in conventional/effector T cells have the opposite effect on Treg and as mTOR activity is differentially regulated between DC subsets, a combinatorial drug therapy can govern activation or inhibition of immune responses (Figure 1). Drug dosing is a major factor that determines whether the response tilts to tolerance.

The mTOR complex is a conserved serine/threonine protein kinase that controls several anabolic processes required for cell growth and proliferation. Consistent with its central role in cell proliferation, several upstream and downstream effectors of mTOR are dysregulated in chronically proliferating malignant cells. The frequency of PI3K/Akt/mTOR hyperactivation in cancer has led to the development of a plethora of inhibitors of this pathway. The commonly used immunosuppressant rapamycin, a macrocyclic triene polyketide, or its analogs (rapalogs) target mTORC1 in tumor cells and are undergoing trials for the treatment of multiple types of cancer. Rapamycin may also promote tumor regression through its ability to inhibit primary and metastatic tumor growth by antiangiogenesis, via the inhibition of VEGF expression. However, the immune suppressive effects of rapamycin might accentuate rather than halt tumor growth. Tumor infiltrating Treg have been associated with a poor prognosis in many non-inflammatory cancers, which are already in an immunosuppressive state. Hence, immunotherapy aims to strengthen the body's immune response to tumors. Conceptually, the combination of chemotherapeutic and immune stimulatory agents should be attractive. Flt3L is being developed to increase T cell activation indirectly via expansion of DC (and to expand NK cells). This growth factor augments the proliferation of DC and other immune cells that express its receptor (Flt3). Notably, Flt3/Flt3L signaling is critical to the generation and steady state expansion of both the conventional (cDC, CD11c^+^, CD8^+^CD11c^+^) and plasmacytoid (pDC, CD11c_mid-lo_PDCA-1^+^) subsets of DC. Flt3L can expand DC from precursor cells *in vivo* for direct mobilization of DC into the site of tumor but may also expand existing Treg, which may then limit immune responses against the tumor. Nonetheless, a combination of Flt3L, rapamycin, and thymidine kinase has been successfully used in a gliomablastoma model [2]. Interestingly, FltL signaling occurs through the PI3K-mTOR pathway, which is inhibited by rapamycin, raising the question of how these drugs can be combined for therapy.

The outcomes of our study established that Flt3L enhanced specific deletion of effector CD4^+^ T cell and Treg induction when co-administered with antigen and rapamycin and thereby improved tolerance induction to an exogenous therapeutic protein. Rapamycin was required for Treg and ultimately tolerance induction, while Flt3L substantially further increased the frequency of induced Treg. The dose of ramapycin used in these experiments was sufficient to block expansion of cDC but not of pDC, which have a more active mTOR signaling pathway and are thus more resistant to inhibition by rapamycin. In fact, pDC enrichment was likely a major factor in Treg induction as shown by depletion experiments. Antigen presentation to T cells in the presence of rapamycin results in activation induced cell death in conventional/effector CD4^+^ T cells, while Treg can utilize alternative signaling and metabolic pathways and thus be induced and expanded under these conditions. Considering all these points, several conclusions can be made on the effect of drug dosing. Intermediate doses of rapamycin block cDC by Flt3L and promote Treg induction and are therefore useful for tolerance induction but may abrogate the desired immune enhancing effects in cancer therapy. High doses of rapamycin hinder expansion of pDCs and may not be as effective in Treg induction. Depletion of pDCs could be a useful tool to limit Treg induction and thus enhance immunity, which is directly supported by data in a breast cancer model [3]. The effect of Flt3L dosing in this balance has not yet been studied but is another likely factor. In addition, the context in which the drugs are administered could influence the outcome. For example, Flt3L can be administered as a polypeptide or expressed from a viral or other gene transfer vector [2].

Treg consistently express the co-stimulatory molecule glucocorticoid-induced TNFR family related gene (GITR), which binds to a ligand that is expressed by pDC and other cell types. Hence, soluble forms of GITR-L have been developed to lift suppression by Treg, thereby enhancing cancer immunotherapy. However, we expanded functional Treg *in vivo* using an Fc fusion of GITR-L that could also be used synergistically with rapamycin for Treg induction [1, 4]. These examples highlight that the same molecular targets may enhance or weaken Treg or effector T cell responses, illustrating the challenge of tipping the balance of the immune response in the desired direction.
